# Exploring the Role of Prey Availability as a Driver of Seasonal Shifts in Local Distribution of Vipers (*Trimeresurus* spp)

**DOI:** 10.1002/ece3.73791

**Published:** 2026-07-05

**Authors:** Alexander H. Murray, Gregory G. Pandelis, Morgan A. Page, Tanagrit Sumpanpa, Panupong Thammachoti Charunrochana, Jesse M. Meik

**Affiliations:** ^1^ Department of Biology Tarleton State University Stephenville Texas USA; ^2^ Amphibian and Reptile Diversity Research Center, University of Texas at Arlington Arlington Texas USA; ^3^ Department of Biology Marshall University Huntington West Virginia USA; ^4^ Department of Biology Faculty of Science, Mahidol University Bangkok Thailand; ^5^ Department of Biology Chulalongkorn University Bangkok Thailand

**Keywords:** amphibian, monsoon, predation, reptile, species interactions, tropics

## Abstract

Predator–prey interactions play a central role in shaping ecological communities, yet how these dynamics shift seasonally in tropical systems remains poorly understood. In this study, we examine how seasonal variation in precipitation and prey availability influence the distribution and abundance of arboreal pitvipers (*Trimeresurus* spp.) in northern Thailand. Using repeated transect surveys across wet and dry seasons, we found that *Trimeresurus* were restricted to streamside habitats during the dry season but expanded their local distribution to include adjacent hillsides during the wet season. Amphibians, a primary prey group for *Trimeresurus*, showed a similar seasonal pattern—highly concentrated near streams during the dry season but dispersed during the wet season. However, snake abundance did not track overall amphibian abundance. Instead, *Trimeresurus* spp. were more closely associated with the presence of rhacophorid tree frogs only, exhibiting significantly higher abundance in areas where these frogs occurred. These findings highlight the importance of prey identity, not just abundance, in shaping predator distribution, and demonstrate how seasonal shifts in prey availability can structure predator spatial ecology in tropical forests.

## Graphical abstract

We investigated how seasonal shifts in precipitation and prey availability affect the distribution of arboreal pit vipers (*Trimeresurus* spp.) in northern Thailand. *Trimeresurus* were confined to streamside habitats during the dry season but expanded into hillsides during the wet season, mirroring amphibian movements. Notably, snake abundance was best explained by the presence of Rhacophorid tree frogs rather than total amphibian abundance, emphasizing the importance of prey identity in shaping predator spatial ecology.

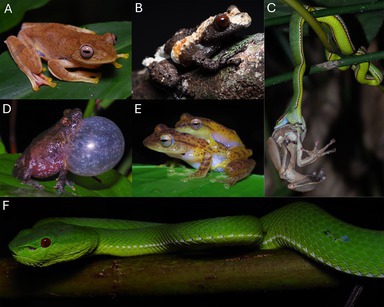

## 1 Introduction

Predator–prey interactions are fundamental to ecological processes, and decades of research have shown that prey availability influences predator abundance, behavior, and habitat use (Elton and Nicholson 1942; Madsen and Shine 1996; Stenseth et al. 1997; Stenseth et al. 1998; Hopcraft et al. 2005; Tutterow et al. 2021). Snakes are obligate carnivores, and their activity, reproduction, and spatial behavior have all been shown to closely track prey availability (Luiselli 2005; Brown and Shine 2007; Baxley and Qualls 2009). Dietary divergence among sympatric species is common (Luiselli 2006; Luiselli et al. 2006; Goodyear and Pianka 2008), and differences in diet have played an important role in driving ecological divergence and evolutionary diversification within this hyper‐diverse squamate clade (Colston et al. 2010; Sherratt et al. 2018; Grundler and Rabosky 2021; Barends and Maritz 2022).

Snakes are especially diverse in the tropics where ecological specialization on prey is common (Arnold 1972; Hoso et al. 2007; Barends and Maritz 2022). While tropical regions tend to be more thermally stable throughout the year than temperate regions, they often exhibit strong seasonal variation in precipitation. As such, seasonal effects on tropical snakes may be driven more by variation in prey availability than by direct climatic stress. Yet, snake ecology in tropical forests remains poorly studied, perhaps due to low detection rates (Asad et al. 2021) and logistical challenges. However, the few studies available suggest that tropical snakes often respond to seasonal changes in prey distribution and abundance (Madsen and Shine 1996; Fonseca et al. 2021).

One common prey group often targeted by tropical snakes is amphibians, particularly anurans (Vitt 1983; de Albuquerque et al. 2010; Giri et al. 2019). Excluding caudates, amphibians reach highest species richness in wet tropical forests (Buckley and Jetz 2007). In tropical forests, amphibian activity and abundance are temporally cyclical and fluctuate with rainfall; however, patterns across studies, regions, and species are inconsistent (Toft 1980; Aichinger 1987; Duellman 1995; Watling and Donnelly 2006; Dubos et al. 2019). Even when amphibians remain active in both the dry and wet seasons, they may shift their local distribution to manage lower humidity levels, often congregating near permanent water bodies (Aichinger 1987) or perching at lower heights (Basham and Scheffers 2020). The importance of anurans in the diet of some tropical snakes in an assemblage was recently demonstrated in Panama, where an outbreak of *Batrachochytrium dendrobatidis* infection caused significant amphibian declines with notable ripple effects on snake diversity and abundance (Zipkin et al. 2020).

**FIGURE 1:**
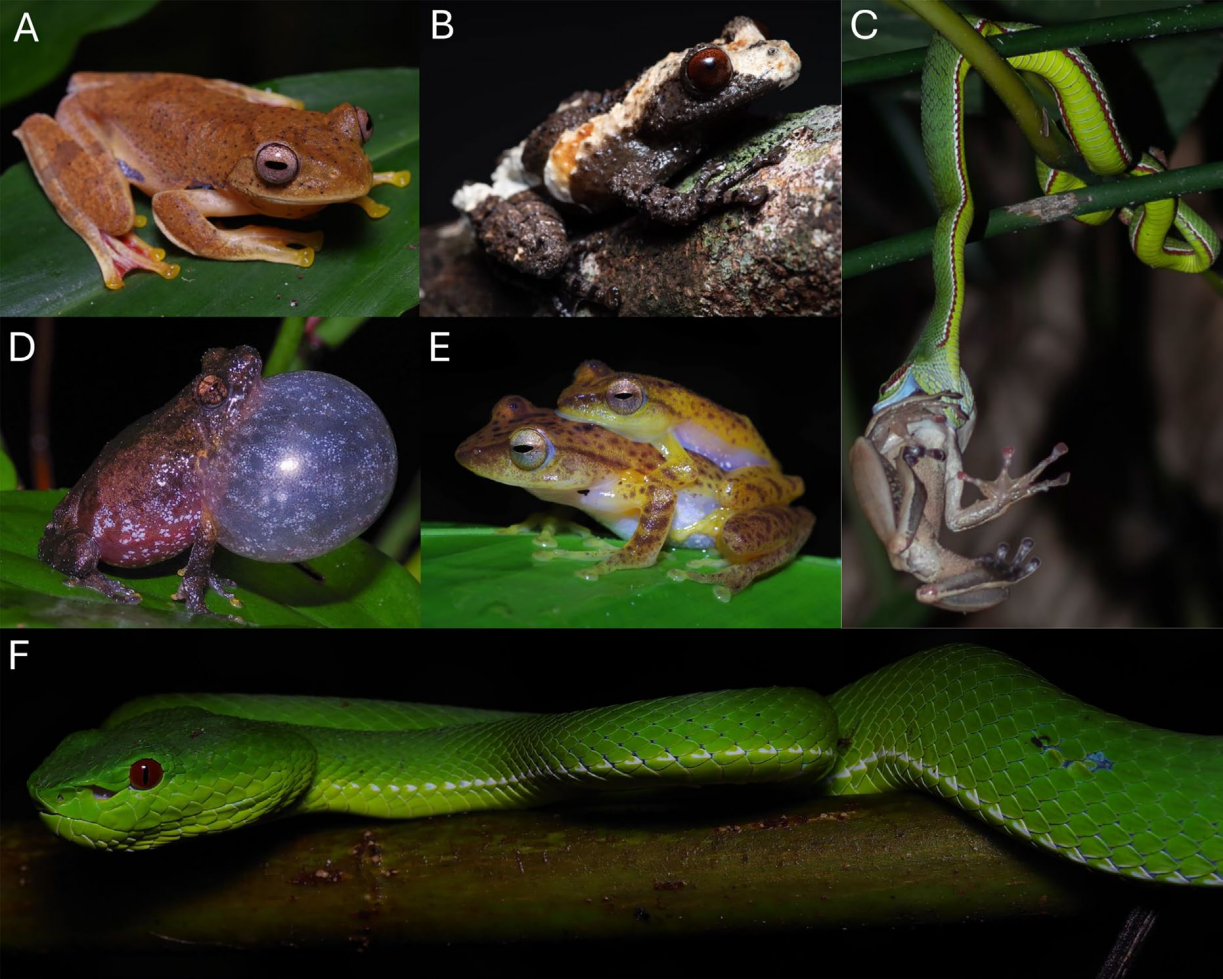
Examples of Rhacophorid frogs from Northern Thailand which were observed on transect and the most common *Trimeresurus* species in Northern Thailand, *Trimeresurus lanna*. (A) *Rhacophorus rhodopus* , (B) *Theloderma albopunctatum* , (C) Small male *Trimeresurus lanna* attempting to eat a large *Polypedates* sp. This was observed ~2 m from stream while surveying transect during February, the *Trimeresurus* was unable to swallow the frog and dropped it. (D) *Raorchestes* sp., (E) *Gracixalus seesom* , (F) *Trimeresurus lanna*.

In this study, we focus on mainland Southeast Asia, a region characterized by intense summer monsoons. Despite a prolonged dry season, these forests harbor exceptional herpetofaunal diversity (Poyarkov et al. 2021; Poyarkov et al. 2023), though this diversity remains poorly studied compared to analogous forested regions in the Neotropics. Arboreal pitvipers of the genus *Trimeresurus* (Figure 1c,f) are widespread and often relatively abundant in mesic forests of southeastern Asia, and are known to feed disproportionately on frogs (Orlov 1997; Creer et al. 2002).

Here we assess how seasonality impacts the local distribution and abundance of the highland *Trimeresurus* assemblage in northern Thailand. Given their preference for anuran prey, we test the hypothesis that seasonal changes in the distribution and abundance of anuran prey will drive changes in the local distribution and abundance of *Trimeresurus*. Specifically, we ask whether *Trimeresurus* abundance tracks overall anuran abundance or is more strongly associated with specific anuran families, which could indirectly shed light on the importance of specific groups driving the diet and indirectly seasonal habitat use in this arboreal pitviper group.

## 2 Methods

### 2.1 Data Collection

Our study focused on montane forests in northern Thailand across five mostly isolated ranges, at elevations ranging from 500 to 1700 m, a region strongly influenced by summer monsoons. Montane forests in northern Thailand receive an annual average rainfall of 1200–1600 mm, with more than 80% of precipitation occurring between May and October (Masud et al. 2016). The landscape is characterized by deciduous lowlands and evergreen highlands, and forest canopies often exceed 30 m in height.

We performed repeat (i.e., spatially and temporally replicated) visual surveys for each site during both the dry season (February to March) and wet season (July to September) in 2024 (Figure S1 and Table S1). For each site (e.g., mountain range), we designated four transects in a consistent spatial configuration to the extent possible: two transects were parallel to streams in riparian vegetation and two transects were positioned away from streams on adjacent hillsides. All transects were located a minimum of 200 m in distance from each other. We aimed to minimize the differences between transects across sites by surveying primary forests and perennial streams of similar order (~2 m width). Forest sites were primarily evergreen forest, and canopy cover varied minimally between the wet season and dry season (> 85% similarity across seasons). Transect surveys consisted of an observer walking at a slow (~1 m/min) pace along a delineated straight line for 100 m through consistent habitat while visually searching all substrates for reptiles and amphibians. A majority of observations occurred within 5 m on either side of the transect line, producing an approximate survey area of roughly 1000m^2^, or 0.1 ha, per transect. Individual surveys were conducted both during the day and night and took 120 min to complete. Given that snakes such as *Trimeresurus* are best detected at night, we included only results from night surveys for this study. Rest time between surveys was consistent across transects as we surveyed each transect both day and night for three consecutive days. We recorded relative humidity and ambient temperature during survey periods using a Kestrel Drop3 environmental data logger. All animals were photographed for *post hoc* confirmation of species identifications.

### 2.2 Analysis

To test whether *Trimeresurus* shifted in abundance and local distribution between seasons, we used a hierarchical modeling approach where detection probability is conditioned on count data. This framework, known as *N*‐mixture modeling, allows for estimates of abundance corrected for variation in detection probability without the requirement of identifying individuals across surveys. Moreover, estimates of model parameters can be refined by incorporating covariates for both survey (i.e., detection) and transect (i.e., abundance) submodels. We analyzed the data at the genus level rather than at the species level for *Trimeresurus*, as *Trimeresurus gumprechti* and *Trimeresurus lanna* (Idiiatullina et al. 2024) may be sympatric at one of our study sites and detailed examination is required for conclusive identification. Observations were primarily of *Trimeresurus lanna* but we also observed *Trimeresurus guoi* (Chen et al. 2021) and possibly *Trimeresurus gumprechti* .

To test the hypothesis that *Trimeresurus* abundance was influenced by frog abundance, we included frog counts as a covariate in our abundance model. We used the average number of frogs observed per survey period on each transect as our anuran abundance variable to account for sites with different numbers of survey periods. After initial model fits with total amphibian abundance as a covariate, we tested whether *Trimeresurus* abundance was impacted by occurrence of different anuran families by including presence or absence of each family on a transect in a given season. Although the impact of anurans and specific anuran families was our primary interest, we also evaluated models with elevation included as a covariate in the transect submodels and environmental variables (ambient temperature, relative humidity) and individual observers as covariates in the survey submodels, allowing the impact of environment and individuals to be modeled explicitly in detection probabilities. Models were fit in a Bayesian framework using a custom Markov chain Monte Carlo (MCMC) approach as implemented in the spAbundance package in R (Doser et al. 2024). We used a Poisson distribution for all models, and default priors and initial values for abundance and detection parameters. We ran three chains each with 16,000 samples, a burn in rate of 20,000 and a thinning rate of 20, to obtain a total of 21,000 posterior samples. Models were run until convergence was achieved, assessed with an rhat value < 1.1 and sufficient ESS. We compared model performance using WAIC values calculated in R.

To assess anuran abundance as a function of seasonality we performed Generalized Linear Mixed Effects models using the package ‘glmmTMB’ (Mollie et al. 2017) in R. We used the sum of anuran species counts across all surveys of a transect within a season as our response variable. We included an interaction effect between season and habitat category (streamside vs. hillside) to allow us to assess if anuran counts changed across the local landscape between seasons. We accounted for unequal sampling effort and variation in elevation by including number of surveys and elevation as fixed effects within our model. We controlled for sites and individual transects as random effects. Models were run using a negative binomial distribution.

## 3 Results

We surveyed 40 unique transects a total of 331 times combined across both seasons, 212 in the dry season and 119 in the wet season (Table S2). We observed 1487 frogs during surveys in the dry season and 671 in the wet season, for a total of 2158 observations. Number of *Trimeresurus* observed on transects in a single survey period ranged from 0 to 6 with a total number of 107 observations, 66 in the dry season and 41 in the wet season. Relative humidity was much greater in the wet season than in the dry season on both stream and hillside transects and during the wet season both the day and night average relative humidity was greater than 90% (Figure S2). In the dry season relative humidity was higher near streams than on hillsides during both the day (59.7% vs. 41.6%) and night (76.8% vs. 58.6%). Temperature was more consistent in the wet season compared to the dry season with daytime and nighttime temperatures during surveys averaging between (21.0°C and 22.8°C) on both streams and hillsides (Figure S2). During the dry season hillside transects were typically warmer than stream transects, both during the day (26.0°C vs. 23.3°C) and night (21.0°C vs. 18.4°C).

**FIGURE 2:**
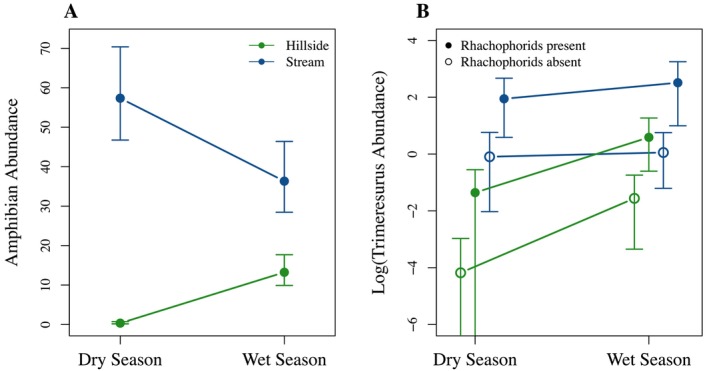
(A) Predicted anuran abundance on transects surveyed between the two seasons, error bars represent standard error. (B) Log of predicted *Trimeresurus* abundance on transect, error bars represent the 83% CRI.

Anuran community abundance was significantly influenced by season, with notable interactions between season and habitat category (Figure 2a and Table 1). During the dry season, anurans were almost exclusively found near streams in high numbers, whereas in the wet season, they were observed both near streams and on hillsides in moderate numbers. Anuran abundance on hillsides was significantly higher in the wet season compared to the dry season, although abundance in streams was lower during the wet season (Figure 2 and Table 1). In the dry season, anurans were observed on only 2 out of 18 hillside transects, with a total of 3 individuals recorded—2 of which were found following the only rainfall over the preceding 2 months. In contrast, during the wet season, anurans were found on 10 out of 12 hillside transects, and they were present on all stream transects during both seasons.

**TABLE 1:** Summary of fixed effects from model anuran abundance model. We denote whether each parameter is significant *p* < 0.1, *p* < 0.01 (**), *p* < 0.001 (***).

Parameters	Estimate	Standard error	*p*
(Intercept)	−2.29535	1.03224	*
Season (wet)	3.66438	0.7601	***
Stream	5.13114	0.73422	***
Elevation	−0.22136	0.19199	
Elevation^2^	0.04481	0.11477	
Survey number	0.21493	0.11567	
Season (wet): stream	−4.12096	0.78077	***

During both seasons *Trimeresurus* abundance was significantly higher near streams compared to hillsides (Figure 2b and Tables S2 and S3). However, during the dry season, *Trimeresurus* was not observed from any of the 18 hillside transects but was present on more than 50% (10/18) of stream transects. In the wet season, *Trimeresurus* was found on 8 out of 11 stream transects and on 4 out of 12 hillside transects (4/9 of which were surveyed 6 times). Detection probability was relatively high and did not differ significantly between the seasons (dry season: mean = 0.44, 25%–75% CRI = 0.35–0.59; wet season: mean = 0.33, 25%–75% CRI = 0.25–0.55). Relative humidity had no impact on detection probability and temperature only significantly impacted detection probability in the dry season, with lower detection probability on warm nights (parameter estimate logit scale mean = −0.99, 2.5%–97.5% CRI = −1.93, −0.07). *Trimeresurus* perch height between the wet and dry seasons was not significantly different (mean dry = 289 cm, mean wet = 198 cm, *p* = 0.14, perch heights were measured with tape measures).

*Trimeresurus* abundance was unimpacted by total anuran abundance and we found no significant relationship between the two. Models including anuran abundance performed worse than those without for both the wet and dry season (Table 2). Of the six frog families included in models, only the presence or absence of rhacophorids on transects significantly affected *Trimeresurus* abundance (Table 2; Tables S2 and S3). *Trimeresurus* abundance was significantly higher on transects with positive detections for rhacophorid frogs (Figure 2; Tables S2 and S3). In the wet season, streams and hillsides with tree frog detections had higher *Trimeresurus* abundance compared to transects without tree frog detections (Figure 2b). However, there was no significant difference in *Trimeresurus* abundance between streams and hillsides with rhacophorid frog detections during the wet season (Figure 2). Estimates for abundance of *Trimeresurus* were highest for stream transects in the wet season that contained rhacophorid frogs, where models predicted a mean of 12.3 snakes per transect (25%–75% CRI = 4.4–12.8), or roughly 123 snakes/ha, whereas stream transects without rhacophorids were predicted to have only 1.1 snakes per transect (25%–75% CRI = 0.5–1.2), or roughly 11 snakes/ha.

**TABLE 2:** Model comparison from wet and dry season. Models with an Amphibian family name included were run with occurrence of that family on transect during a season as 0 or 1 for presence or absence.

Model	Dry season		Wet season	
	WAIC	ΔWAIC	WAIC	ΔWAIC
Elevation + Elevation^2^	114.1	18.2	120.9	9.2
Stream + Elevation + Elevation^2^	96.9	1.0	119.7	8.0
Stream + Amphibian abundance + Elevation + Elevation^2^	98.1	2.2	121.3	9.6
Stream + Bufonidae + Elevation + Elevation^2^	96.8	0.9	121.1	9.4
Stream + Dicroglossidae + Elevation + Elevation^2^	97.2	1.2	119.4	7.7
Stream + Megophryidae + Elevation + Elevation^2^	98.2	2.2	118.1	6.4
Stream + Microhylidae + Elevation + Elevation^2^	97.2	1.3	121.2	9.5
Stream + Ranidae + Elevation + Elevation^2^	98.5	2.6	120.4	8.7
Stream + Rhacophoridae + Elevation + Elevation^2^	95.9	0.0	111.7	0.0

Across both seasons, transects with rhacophorid frogs were much more likely to also have *Trimeresurus* (66.7%, 16/24) compared to transects without rhacophorids (17.1%, 6/35). In the dry season, rhacophorid frogs were absent from all hillside transects, and *Trimeresurus* were also absent from these transects. In contrast, during the wet season, rhacophorid frogs were found on 7/12 hillside transects, and *Trimeresurus* were present on 4 of those transects; no hillside transects without rhacophorid frog observations had *Trimeresurus* observations. For stream transects in the dry season, rhacophorid frogs were present on 13/18, and *Trimeresurus* were found on 8 of these. In the wet season, rhacophorid frogs were present on 4/11 stream transects, with *Trimeresurus* present on all four of these (Table S4).

## 4 Discussion

The monsoon had clear effects on both anuran and arboreal pitviper communities in northern Thailand. While prior studies in tropical Asia have documented seasonal turnover in amphibian assemblages (Ohler et al. 2000; Galoyan et al. 2018), increased species richness during the wet season (Singh Mehra et al. 2021) and elevated encounter rates across the landscape (Crane et al. 2018), few have quantified how amphibian distributions shift at local spatial scales between seasons. Our findings reveal a seasonal contraction of anuran local distributions, with frogs almost entirely restricted to streamside habitats during the dry season and broadly dispersed throughout the forest during the wet season (Figure 2a). *Trimeresurus* mirrored this shift, occupying only streamside areas in the dry season and expanding their distribution in tandem with their amphibian prey during the wet season. Interestingly, *Trimeresurus* abundance was not directly correlated with overall frog abundance. Instead, the presence of rhacophorid tree frogs specifically was a stronger predictor of *Trimeresurus* abundance on our transects; across both habitat types and seasons *Trimeresurus* abundance was higher where tree frogs were present. Our results suggest that rhacophorid local distribution plays a role in shaping the local distribution of *Trimeresurus*, and that seasonal shifts in snake occupancy may be a response to changes in prey availability across the landscape rather than just a direct response to seasonal changes in microclimate.

The strong association between *Trimeresurus* and rhacophorid tree frogs is not surprising, given their arboreal habitat use and that other species of *Trimeresurus* are known to prefer tree frogs over other frog species (Orlov 1997; Creer et al. 2002; Yang et al. 2024). Yet the link between the seasonal distribution of *Trimeresurus* and their preferred prey has previously not been well established. Our findings parallel those from a study in India that documented seasonal shifts in abundance in the closely related arboreal viper genus *Craspedocephalus*, which was concentrated near water bodies during the dry season and more widely dispersed during the monsoon (Sawant and Jadhav 2013). Although data on shifts in amphibian data were not presented, the similarity in spatial patterns suggests that prey availability might also be a key underlying driver.

Snake detections were similar across seasons, but we observed *Trimeresurus* crossing roads only during the wet season, suggesting that long‐distance movement may be constrained to periods when prey is more broadly distributed and climatic conditions are favorable. This observation is consistent with findings from a recent study that found that rainfall was positively correlated with the number of observations of *Trimeresurus albolabris* and *T. macrops* in Thailand (Ratnarathorn et al. 2024). One interesting possibility is that by specializing on prey that is heavily impacted by the seasonality of precipitation, *Trimeresurus* reduces energy expenditure on mate searching during times of year, in this case the dry season, when prey and snakes are aggregated in proximity to streams. Consistent with this idea, we observed both predation and mating behavior along streams during the dry season, including a confirmed mating event of *T. lanna* on 2 March 2024. However, additional observations and data will be necessary to evaluate whether dry‐season mating is generalizable for the species in our study.

Few multi‐season studies have examined other arboreal vipers, but available evidence reinforces the role of prey population dynamics in shaping snake ecology. In Brazil, *Bothrops bilineatus* , an arboreal species that preys on frogs, was found to be most abundant during the dry season, with its distribution more closely tied to the presence of specific hylid frogs than to total amphibian abundance (Fonseca et al. 2021). Conversely, *Atheris squamigera*, an African arboreal viper that does not primarily consume amphibians, showed a pronounced decline in activity during the dry season (Luiselli et al. 2000), underscoring the importance of prey type in determining seasonal behavior. Beyond vipers, the seasonal distributions of many tropical snakes are known to be strongly influenced by prey availability. For instance, in northern Australia, *Liasis fuscus* (water pythons) makes long distance movements, up to 12 km, to track the availability of high quality prey (Madsen and Shine 1996). These examples, along with our findings, highlight a broader pattern: while snake responses to precipitation regimes are highly variable across taxa (Henderson and Hoevers 1977; Mesquita et al. 2012), prey availability consistently emerges as a key ecological driver.

*Trimeresurus* spp. are an excellent candidate group for ecological studies of tropical snakes, as they are diverse, sometimes occur at high densities as demonstrated here (> 100 snakes/ha), and are relatively easy to detect (detection probability > 33%). Ultimately, this study contributes to a growing recognition that predator–prey dynamics are crucial for understanding seasonal shifts in habitat use in otherwise seemingly stable tropical ecosystems. Future studies may wish to include radio telemetry, mark recapture, and diet analysis to better understand drivers of seasonal differences in local distribution and confirm our conclusions on the relationship between rhacophorids and *Trimeresurus*. To develop broader ecological generalizations about species abundance and diversity in the tropics, it is essential to expand the collection of fine‐scale, systematically gathered community data. Increasing the number of studies that span multiple seasons will be especially critical for uncovering how temporal variability, such as precipitation seasonality, shapes ecological patterns and processes. Such efforts are vital for advancing a more mechanistic understanding of tropical biodiversity.

## Author Contributions

**Alexander H. Murray:** conceptualization (lead), data curation (lead), formal analysis (lead), investigation (lead), methodology (lead), visualization (lead), writing – original draft (lead), writing – review and editing (lead). **Gregory G. Pandelis:** investigation (supporting), writing – review and editing (supporting). **Morgan A. Page:** investigation (supporting), writing – review and editing (supporting). **Tanagrit Sumpanpa:** investigation (supporting), writing – review and editing (supporting). **Panupong Thammachoti Charunrochana:** investigation (supporting), resources (supporting), writing – review and editing (supporting). **Jesse M. Meik:** conceptualization (supporting), supervision (lead), writing – review and editing (supporting).

## Conflicts of Interest

The authors declare no conflicts of interest.

## Supporting information


**Figure S1:** Distribution of Sampling locations in Northern Thailand, numbers on map correspond to sites in Table S1. Elevation strongly influences forest type, and in general below 800 m forests in northern Thailand are often deciduous, thus the color gray for the lowlands.
**Figure S2:** (A, B) Average relative humidity and (C, D) average temperature from transects between 11 and 1200 m in elevation, where the majority of transects occurred.
**Table S1:** Number of surveys by location across the dry season and wet season in Northern Thailand. Site number corresponds to number on map above in Figure S1.
**Table S2:** Model summary of the best model for the dry season, corresponding to model 9 in Table 2.
**Table S3:** Model summary of the best model for the wet season, corresponding to model 9 in Table 2.
**Table S4:** Summary of presence and absence of Rhacophorid frogs and *Trimeresurus* on transects by season.

## Data Availability

Data and code required for all analyses in this paper are contained in the supplemental files can be found at https://doi.org/10.6084/m9.figshare.32682687.
