# Supplementary material for: Exploring the Role of Prey Availability as a Driver of Seasonal Shifts in Local Distribution of Vipers (Trimeresurus spp)
Source: Ecol Evol. 2026 Jul 5;16(7):e73791. doi: 10.1002/ece3.73791 (PMC13333253; doi:10.1002/ece3.73791)
Supplement: Supplementary file 1 — Figure S1: Distribution of Sampling locations in Northern Thailand, numbers on map correspond to sites in Table S1. Elevation strongly influences forest type, and in general below 800 m forests in northern Thailand are often deciduous, thus the color gray for the lowlands. Figure S2: (A, B) Average relative humidity and (C, D) average temperature from transects between 11 and 1200 m in elevation, where the majority of transects occurred. Table S1: Number of surveys by location across the dry season and wet season in Northern Thailand. Site number corresponds to number on map above in Figure S1. Table S2: Model summary of the best model for the dry season, corresponding to model 9 in Table 2. Table S3: Model summary of the best model for the wet season, corresponding to model 9 in Table 2. Table S4: Summary of presence and absence of Rhacophorid frogs and Trimeresurus on transects by season. [file ECE3-16-e73791-s001.docx]

Supplemental Information

**Figure S1.** Distribution of Sampling locations in Northern Thailand, numbers on map correspond to sites in Table S1. Elevation strongly influences forest type, and in general below 800m forests in northern Thailand are often deciduous, thus the color grey for the lowlands.

**Table S1.** Number of surveys by location across the dry season and wet season in Northern Thailand. Site number corresponds to number on map above in Figure S1.

**Figure S2. A,B** Average relative humidity and **C,D** average temperature from transects between 11-1200m in elevation, where the majority of transects occurred.

**Table S2**. Model summary of the best model for the dry season, corresponding to model 9 in Table 2.


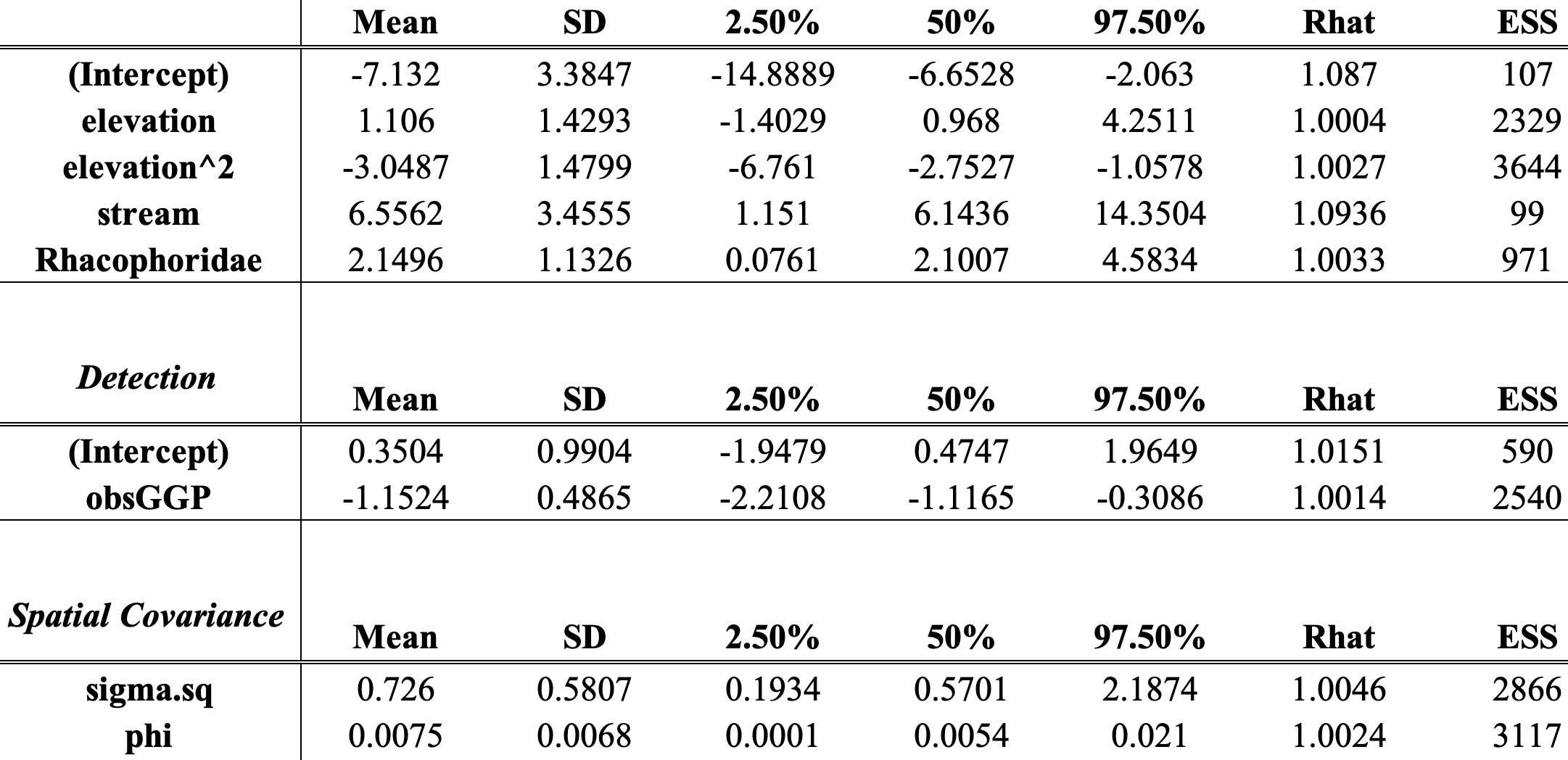


**Table S3**. Model summary of the best model for the wet season, corresponding to model 9 in Table 2.


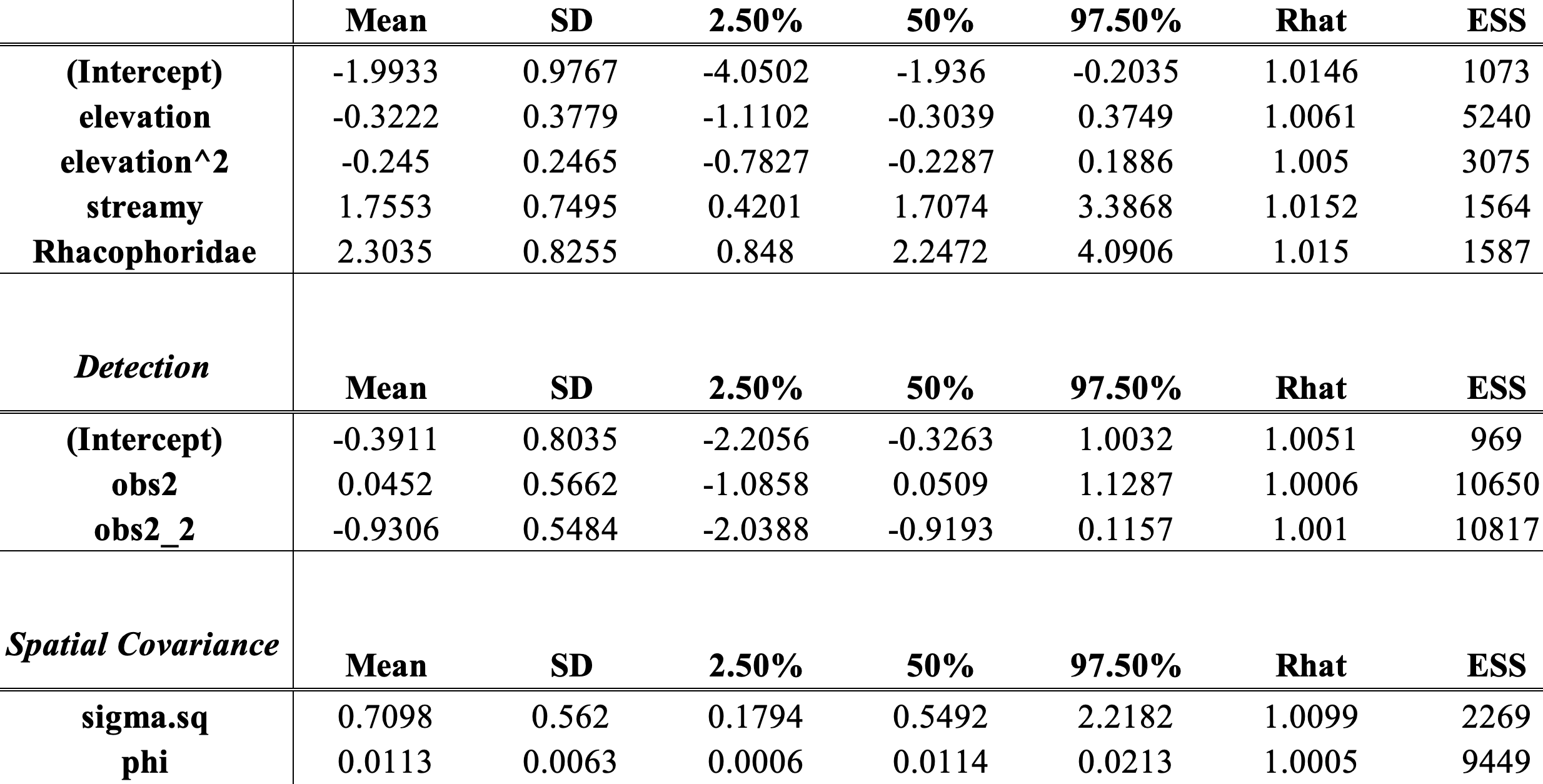


**Table S4.** Summary of presence and absence of Rhacophorid frogs and *Trimeresurus* on transects by season.
